# The identification of needs and development of best practice guidance for the psychological support of frontline healthcare workers during and after COVID-19: A protocol for the FLoWS project

**DOI:** 10.12688/hrbopenres.13117.2

**Published:** 2021-01-29

**Authors:** Jack Flynn, Laura O'Connor, Michelle Hanlon, Giacomo Bellani, Maya Contreras, Anne Doherty, Hannah Durand, Elaine Fallon, Clare Gormley, John Laffey, Gerry Molloy, Kiran Sarma, Maria Grazia Strepparava, Selena Russo, Jane Walsh, Brian E. McGuire

**Affiliations:** 1 School of Psychology, National University of Ireland, Galway, Ireland; 2 School of Medicine and Surgery, University of Milano-Bicocca, Milan, Italy; 3 San Gerardo Hospital, Monza, Italy; 4 University Hospital Galway, Galway, Ireland; 5 School of Medicine, National University of Ireland, Galway, Ireland; 6 Saolta University Health Care Group, Galway, Ireland; 7 Health Service Executive West, Galway, Ireland

**Keywords:** COVID, Pandemic, Frontline workers, Psychological distress, Support needs, Best practice guidelines

## Abstract

Recent estimates suggest that up to 34% of frontline workers in healthcare (FLWs) at the forefront of the COVID-19 pandemic response are reporting elevated symptoms of psychological distress due to resource constraints, ineffective treatments, and concerns about self-contamination. However, little systematic research has been carried out to assess the mental health needs of FLWs in Europe, or the extent of psychological suffering in FLWs within different European countries of varying outbreak severity. Accordingly, this project will employ a mixed-methods approach over three work packages to develop best-practice guidelines for alleviating psychological distress in FLWs during the different phases of the pandemic. Work package 1 will identify the point and long-term prevalence of psychological distress symptoms in a sample of Irish and Italian FLWs, and the predictors of these symptoms. Work package 2 will perform a qualitative needs assessment on a sample of Irish and Italian FLWs to identify sources of stress and resilience, barriers to psychological care, and optimal strategies for alleviating psychological distress in relation to the COVID-19 pandemic. Work package 3 will synthesise the findings from the preceding work packages to draft best practice guidelines, which will be co-created by a multidisciplinary panel of experts using the Delphi method. The guidelines will provide clinicians with a framework for alleviating psychological distress in FLWs, with particular relevance to the COVID-19 pandemic, but may also have relevance for future pandemics and other public health emergencies.

## Introduction

In late 2019, public health authorities in China informed the World Health Organisation (WHO) of a cluster of pneumonia-like presentations detected in the city of Wuhan. The WHO subsequently named the virus ‘severe acute respiratory syndrome coronavirus 2’ (SARS-CoV-2), and the illness that it causes ‘coronavirus disease 2019’ (COVID-19) ( Peng *et al*., 2020). By July 15 ^th^, 2020, over 13 million people had been diagnosed with the virus worldwide and more than 570,000 people had died ( European Centre of Disease Control and Prevention, 2020).

A significant proportion of those who experience symptoms (55–60% estimated by Oran & Topol, 2020) develop serious disease, including acute respiratory distress syndrome (9.4%), acute cardiac injury (5.8%) and acute kidney injury (2.1%; Hu *et al*., 2020). Due to the lack of effective antiviral therapies ( Sanders *et al*., 2020), symptomatic patients admitted to hospitals, particularly older adults and those with underlying health conditions, require lengthy intensive care unit stays with invasive mechanical ventilation, nasal cannula, and vasopressor treatment ( Bhatraju *et al*., 2020). Although the mortality rate for all infections is relatively low at 3.2%, case studies have identified mortality rates between 32% and 50% for patients admitted to hospital ( Bhatraju *et al*., 2020; Docherty *et al*., 2020). 

Those on the frontlines of the COVID-19 healthcare response, including doctors, nurses and paramedics, have had to quickly adapt to a novel, highly transmittable, and lethal disease. These frontline workers (hereafter referred to as FLWs) are at risk of developing psychological distress due to a range of COVID-related experiences, which includes reduced response efficacy, existential threat, and the fear of contracting the virus and transmitting it to others ( Spoorthy *et al*., 2020). This aligns with recent meta-analytic findings based on research involving more than 33,000 FLWs responding to COVID-19, and which suggested high levels of depression (22.8%), anxiety (23.2%) and insomnia (34.32%) ( Pappa *et al*., 2020). In terms of moderate and severe symptoms, pooled prevalence estimates of 16.18% were identified for depression and 6.88% for anxiety ( Pappa *et al*., 2020). The severity of these symptoms has been shown to vary based on demographic and occupational factors, with healthcare workers of female gender, intermediate professional status, and with low social support, yielding the highest odds of experiencing elevated psychological distress ( Spoorthy *et al*., 2020). However, these studies were mainly carried out in Asia and therefore the findings may not reflect the proportion or determinants of psychological distress of FLWs internationally, given the cultural differences in experiencing work-related stress ( Györkös *et al*., 2012) and seeking psychological support ( Mojaverian *et al*., 2013), and in provision of resources and healthcare expenditure ( Jakovljevic *et al*., 2019).

In addition, scarcity of resources may lead to FLWs feeling an inability to adequately care for their patients, leading to poorer outcomes or avoidable deaths, which could result in them experiencing moral injury. Moral injury is described as profound psychological distress resulting from actions (or omissions) which violate the moral or ethical code of the affected person ( Litz *et al* 2009). Such actions may include acts of perpetration and acts of omission, and are associated with the development of mental health problems, including depression, post-traumatic stress disorder (PTSD) and anxiety disorders ( Williamson *et al*, 2018). Much of the research in moral injury has been conducted in military personnel, but since the outbreak of COVID-19 there has been concern that this may similarly apply to FLWs ( Williamson *et al*., 2020).

In the context of mass trauma, where multiple individuals have been exposed to the same traumatic event, the advised approach to providing psychological care is to tailor interventions to the unique challenges posed by each specific event ( Reifels *et al*., 2013). As such, identifying novel sources of stress and resilience, perceptions of barriers to psychological care, and groups at risk of psychological distress is essential for tailoring psychological care ( Reifels *et al*., 2013). However, only a small number of studies have attempted to identify common coping mechanisms in FLWs during the COVID-19 pandemic ( Spoorthy *et al*., 2020). One study of Chinese FLWs identified a number of active coping strategies employed by FLWs during the COVID-19 pandemic, including yoga, mindfulness, progressive relaxation, social support from fellow FLWs, self-reflection, and seeking positive emotions through altruism ( Sun *et al*., 2020). Another study of FLWs in Beijing highlighted the use of regular communication with family members and colleagues through video-chat or telephone as the primary coping mechanisms employed ( Cao *et al*., 2020). Again, these studies have all assessed FLWs in an Asian context, and the findings may not reflect the coping mechanisms employed by FLWs internationally.

As psychological distress is a significant contributor to burnout and medical errors in healthcare professionals ( Hall *et al*., 2016), the development of resources to support the mental wellbeing of FLWs is considered paramount to ensure the safety of both FLWs and patients throughout the COVID-19 pandemic ( Santarone *et al*., 2020). Several professional bodies have shared guidelines for alleviating psychological distress in those exposed to crisis situations, which mostly pertain to situations with short exposure periods that affect small proportions of the population, including natural disasters and terrorist attacks ( Te Brake *et al*., 2009). Due to the unprecedented scale, protracted duration, and global reach of the COVID-19 pandemic, these guidelines may not be applicable in this context, and novel research is required to identify the psychological needs of those directly exposed to the pandemic. However, despite the massive public health and economic impact of COVID-19 in Europe ( Nicola *et al*., 2020; Pang *et al*., 2020), limited research has evaluated the prevalence and predictors of psychological distress in European FLWs, the extent to which these symptoms vary between countries of differing outbreak severity, or the psychological needs of FLWs in a European context. This protocol outlines a series of studies aimed at addressing this evidence gap, with the aim of developing best practice guidance for alleviating psychological distress in FLWs during pandemics. Specific research questions were devised by our steering group, aligning with the current available literature as described above. Quantitative data will be collected to assess the experience of psychological distress in a broad sample of FLWs, and a qualitative interview study will explore personal experiences and support needs in more detail. To assess whether differential rates of COVID-19 influences the prevalence of psychological distress symptoms, and the needs required to address these symptoms, both studies will collect data in Ireland and Italy concurrently. All findings will then feed into the development of best practice guidelines, which will be drafted and validated by a panel of national and international stakeholders.

## Methods

This research project will employ a mixed methods approach over three work packages to address the research objectives. Work Package 1, a quantitative survey to be carried out online in Ireland and Italy, will gather data from a broad sample of FLWs to establish the prevalence and determinants of psychological distress in FLWs in both countries, with a follow-up survey to be carried out 6 months later in order to look at delayed effects. Work Package 2, a qualitative study running concurrently with Work Package 1, will conduct interviews with FLWs in both Ireland and Italy pertaining to their mental health needs in the context of the COVID-19 pandemic. Work Package 3 will synthesise the findings from the preceding work packages to draft best practice guidance regarding the alleviation of psychological distress in FLWs. A validation process of the drafted guidelines will follow, using the Delphi method.

### Population

Across all work packages, a FLW will be defined as a person working in healthcare whose role during the pandemic was likely to require repeated contact with patients with COVID-19, whether confirmed or suspected ( Lai *et al*., 2020; Nguyen *et al*., 2020). This is likely to include doctors, nurses, and allied health professionals working in emergency departments, ICUs, and COVID-19 designated facilities and inpatient wards, but does not exclude other departments, primary care facilities, or those who work in the community. It may also include essential non-clinical staff working in these areas, such as technical, administrative, cleaning and catering staff.

Participants for both studies will be recruited through hospital communications to staff (e.g. emails, noticeboards), professional organisations and training colleges, and by asking FLWs to share details with colleagues. Participants are likely to participate in either the online questionnaire or the interview, but may participate in both if they so wish. A truly representative sample of FLWs is unlikely to be reached given the broad range of roles that are eligible to participate, but responses will be monitored and outreach strategies adjusted to avoid significant over- or under-representation of any one subgroup.

### Inclusion Criteria

 Work packages 1 and 2 will utilise the same inclusion criteria, outlined below.

- Worked as a FLW as defined above during the pandemic (whether as part of their normal role or as a temporary redeployment)
- Able to provide consent online
- Sufficiently fluent in English/Italian to be able to take part in an interview and/or an online survey

### Work Package 1: Prevalence and determinants of psychological distress in Irish and Italian frontline workers

The aim of this work package is to determine the proportion of FLWs with elevated symptoms of psychological distress to indicate the magnitude of psychological suffering in Irish and Italian FLWs. This work package also aims to evaluate the extent to which moral injury, demographic and occupational factors predict psychological distress in Irish and Italian FLWs, thereby providing an indication of groups at-risk of psychological distress. To do this, a cross-sectional design will be employed, whereby psychometric measures and questionnaires are administered to Irish and Italian FLWs. Each participant will complete an online survey comprised of psychometric scales to assess symptoms of depression (PHQ-9; Kroenke *et al*., 2001), anxiety (GAD-7; Spitzer *et al*., 2006), insomnia (ISI-7; Bastien *et al*., 2001), post-traumatic stress (PCL-5; Blevins *et al*., 2015), post-traumatic growth (Post-traumatic Growth Inventory; Tedeschi & Calhoun, 1996), adjustment disorder (ADNM-8; Kazlauskas *et al*., 2018), and moral injury (Moral Injury Events Scale; Nash *et al*., 2013). The survey will also include a questionnaire to record age, gender, marital status, country of residence, type of residence, occupation title, professional experience, and type of hospital. Additional areas of interest as suggested by clinicians are perceptions of personal readiness for their usual or redeployed roles, perceived control over the influx of patients, availability and utility of training, institutional support, and impacts on personal life and living arrangements. An additional series of questions will be provided to assess these perceptions. Finally, to ensure that any recommendations made within the guidelines reflect support services that FLWs would realistically avail of, participants will be asked to preferentially rank a series of support services based on the recommendations of clinicians (e.g. psychological support, more PPE) which they either did avail of, or would have availed of, throughout the pandemic. Participants will be asked to rank these support services from being the most to least preferential based on their perceived usefulness for alleviating psychological distress throughout the pandemic. Efforts will be made in the design of the questionnaire to reduce participant burden and ensure time needed to complete is kept to a minimum.

To ensure the sample size is large enough to facilitate multivariate analyses of the range of predictors being assessed (demographics, occupational factors, moral injury), the following formula was used: *N*=100+50 *i*, where *N* and *i* respectively denote the sample size and number of predictors being included in the models ( Bujang *et al*., 2018). Based on approximately 20 coefficients being generated within the models, a minimum of 1,100 participants, recruited across Ireland and Italy, is necessary to yield representative parameter estimates and avoid model over-fitting. This sample size is also sufficient to generate prevalence estimates of the psychological distress symptoms being assessed using the traditional prevalence formula: *N* = *Z* ^2^ *P*(1 − *P*) / *d* ^2^, where the sample size *N* is generated using the conventional values of 95% and 5% for the level of confidence *Z* and precision *d*, respectively, and the prevalence value *P* based on existing estimates (i.e., 22.8% for depression, 23.3% for anxiety, 34.32% for insomnia, 71.5% for distress; Lai *et al*., 2020; Pappa *et al*., 2020). A convenience sample of FLWs will be recruited through health service networks in Ireland and Italy.

While an earlier version of this protocol included plans for a second questionnaire carried out 6 months later, the ever-changing course of the pandemic and the additional burden this would create for FLWs led to this plan being adapted. A second questionnaire will now not be carried out in the course of this project.

***Research questions***. 1. What is the proportion of Irish and Italian FLWs with mild, moderate, and severe symptoms of depression, anxiety, insomnia, and distress at time of assessment and after 6 months? 

2. What are the predictors of depression, anxiety, insomnia, and distress in Irish and Italian FLWs at time of assessment and after 6 months? 

***Data analysis plan***. Descriptive statistics for the total sample will comprise of means with standard deviations for normally distributed continuous variables, medians with interquartile ranges for non-normal continuous variables, and frequencies with proportions for categorical variables. The prevalence of psychological distress symptoms will be determined using the proportion of respondents with either mild symptoms, moderate or severe symptoms in accordance with the standardised cut-off scores for each measure.

To provide a preliminary indication of the determinants of the psychological distress, a series of independent sample t-tests, Mann-Whitney U-tests, and Chi-square tests will be used, where appropriate, to compare the distributions of psychological distress symptoms between each demographic and occupational group. To determine the independent influence of these factors in predicting psychological distress, multiple logistic regressions will be conducted using each psychological distress symptom as a dichotomized outcome variable based on the standardised cut-off score for each measure. For each symptom, one logistic regression will examine correlates of at least mild symptoms, and a second logistic regression will examine the correlates of at least moderate to severe symptoms. Each logistic regression will control for the geographical region where the participants are employed (i.e., Ireland or Italy), thereby indicating whether one cohort is at higher odds of experiencing psychological distress, but also allowing for an analysis of the demographic/occupational predictors of psychological distress independent of geographical location. Each logistic regression will also include the participants’ occupation, whether they are employed in a rural or urban setting, gender, years of professional experience, and marital status. Odds ratios (OR) and 95% confidence intervals (95% CI) will be used to determine whether any of the geographical, occupational, or demographic characteristics of the sample are independently predictive of higher odds of experiencing psychological distress. Exploratory moderation analyses will also be considered to determine whether the influence of predictor variables on the outcome variable varies depending on the status of other variables (e.g., whether the influence of occupational status on psychological distress differs between the Irish and Italian participants), and whether these interactions significantly contribute to the fit of the model using the maximum likelihood method. 

Additional descriptive analyses will also examine the proportion of participants reporting differing levels of readiness for their professional roles during the pandemic, whether they received training, their perceptions of the suitability of the training, whether they received institutional support, and the extent to which the pandemic adversely affected their family life. Finally, the preferentially ranked support services will be examined by determining which services yielded the largest number of participants ranking them as being the most preferential. 

### Work Package 2: Qualitative needs assessment of Irish and Italian frontline workers

The aim of this work package is to qualitatively evaluate FLW’s first-hand accounts of their primary sources of stress and resilience during the pandemic, their perceptions of barriers and facilitators to psychological care, and insights into whether and how psychological services can alleviate stress in FLWs. To do this, a qualitative interpretative design using thematic framework analysis will be employed, using a purposive sampling and snowballing strategy to recruit participants. Interviewees will be sent an invitation with a link to the information form via e-mail. They will then be contacted by a member of the research team who will explain the purpose of the interview and schedule an appointment. In line with social distancing guidelines, electronic consent will be sought from all participants to prevent face-to-face contact. Interviews will take approximately one hour. All interviews will be audio-recorded and transcribed verbatim. To ensure the quality of the research findings, the sample size for the semi-structured interviews will range between 20 to 30 participants, recruited across Ireland and Italy, in line with existing guides on sampling requirements for qualitative interviewing ( Francis *et al*., 2010; Green & Thorogood, 2018; Ritchie *et al*., 2003). The exact number recruited will be determined by the point at which the data reaches saturation ( Guest *et al.*, 2006).

Semi-structured interviews will be conducted with individual participants via phone or a secure web-based platform. Semi-structured interviews were chosen as they are flexible and allow participants to elaborate on issues that they want to speak more about, yet still provide a good structure for comparability ( Jamshed, 2014). Semi-structured interviews also allow the researcher and participant to engage in a dialogue whereby initial questions are modified in the light of the participant’s responses and the investigator is able to probe interesting and important areas that arise. An interviewing topic guide of open-ended questions will be used to flexibly guide the interviews and to minimize variability, and enhance comparability, across the interviews. This interview guide will be developed based on a topic list that was developed from the research questions.

***Research questions***

1. What are the primary sources of stress and resilience for Irish and Italian FLWs during the different stages of the COVID-19 pandemic?
2. According to Irish and Italian FLWs, what are the optimal strategies for alleviating psychological stress in FLWs during the different stages of the COVID-19 pandemic?
3. What, if any, are the perceived barriers for accessing psychological support, and perceived value of these supports, in Irish and Italian FLWs during the different stages of the COVID-19 pandemic?

***Data analysis plan.*** Interview transcripts will be analysed using thematic framework analysis. The framework method, as described by Gale *et al.* (2013), was chosen for this study as it can compare data across cases as well as within individual cases and allows for the inclusion of *a priori* as well as emerging themes. It also provides a highly systematic method for categorizing and organizing qualitative data as the procedure for applying framework analysis consists of seven distinct stages: transcription, familiarization, coding, developing an analytical framework, charting and interpreting (see Hanlon *et al*., 2020 for details on the method). Having a systematic method such as this will be useful to ensure consistency as there will be multiple researchers working on the project. The framework method also produces highly structured outputs of summarised data, which is beneficial for projects such as this where the research team will need to manage a large data set while also obtaining a holistic, descriptive overview of the entire data set.

The researchers are cognisant of the necessity for quality assurance and rigour throughout the data collection, analysis and write up stages of this work package. Each researcher involved in interviewing participants and analysing the data has the necessary education, skills, and experience that is required for conducting high quality qualitative research. Rigour will be established using the criteria of Reliability, Validity, Dependability, and Confirmability. In line with guidelines outlined by Morse *et al*. (2002) and Whittemore *et al*. (2001), this will be achieved with care in the application of research practices to ensure replicability and consistency, providing verbatim transcriptions of the interviews, demonstrating saturation, clearly articulating data analysis decisions, reflexive journaling by the researchers following each interview, providing an audit trail, and demonstrating evidence (i.e. using participant quotes and a synthesis of researchers perspectives) that supports interpretations.

### Work Package 3: Development of best practice guidelines

The aim of this package is to utilise the findings from the earlier work packages to draft best practice guidelines for alleviating psychological distress in FLWs. Researchers with a diverse range of expertise, including psychiatrists, clinical psychologists, health psychologists, physicians, and nurses will all provide input for interpreting the findings and determining how they can be realistically and effectively applied to alleviate psychological distress in FLWs. Qualitative data will be used to identify modifiable sources of stress, methods to overcome perceived barriers to psychological care, and common sources of resilience which can be used to foster effective coping mechanisms in less resilient workers. Quantitative data will be used to identify the most prevalent psychological distress symptoms in FLWs, and the most effective evidence-based treatments for alleviating those symptoms can be recommended accordingly. Through identifying determinants of psychological distress, recommendations can be made concerning which at-risk groups warrant additional attention for psychological care. The extent to which the drafted guidelines are generalized across different cultures (i.e., Irish and Italian) and occupational groups will be dependent on the congruency between the quantitative and qualitative findings of the groups assessed. For example, should certain demographics be more indicative of psychological distress in the Irish cohort, or FLWs in the Italian cohort report different psychological needs, the guidelines will be developed to reflect these differences.

The drafted best practice guidelines will be validated using the Delphi method, whereby a purposively selected panel of national and international stakeholders will review the guidelines and amend any of the content based on their insights. Eligible members of this panel will include providers of psychological support (e.g., clinical psychologists, psychotherapists, psychiatrists) and FLWs who are being targeted for provision of psychological support (e.g., doctors, nurses), to ensure the feasibility and acceptability of implementing suggestions is considered. To ensure the guidelines are applicable to FLWs with varying levels of seniority and differing experiences throughout the pandemic, efforts will be made to recruit both junior and senior FLWs, and FLWs who either remained in their usual position or were re-deployed during to the pandemic (with FLWs as defined as in WP1 and 2 eligible for inclusion). Panel members will be recruited through searching relevant national and international organisations, literature, and recommendations from the project team and Delphi participants. Although there is no standardized sample size for the Delphi approach, a minimum of 10 panel members per area of expertise is recommended to ensure reliability in the group’s judgements ( Okoli & Pawlowksi, 2004). Accordingly, a minimum sample of 10 providers of psychological support and 10 FLWs will be targeted for the panel, although this may be subject to change based on the availability of participants, and the ability to recruit within a short timeframe. 

In accordance with recommendations on how to reach optimal decision making using the Delphi method, all panel members will provide insights anonymously and work autonomously, moderated by a member of the research team with experience in managing discussions acting as a panel facilitator ( Jorm, 2015). The round one Delphi survey will ask panel members to rate their level of agreement with each of the recommendations on a 5-point Likert scale (‘strongly disagree’ to ‘strongly agree’). Open text space for participants to give reasons for their evaluations, and suggestions for changes and additional content will be provided. For each recommendation outlined within the guidelines, a summary statistic will be used to quantify the proportion of panel members that either agree or disagree with the usefulness or practicality of each corresponding strategy, where 80% agreement is required for consensus ( Jorm, 2015). Should a large proportion of panel members disagree with the usefulness or practicality of a proposed strategy, their recommendations will be used to amend such strategies until a consensus is reached, or that strategy is omitted from the final set of guidelines. 

## Ethical considerations

This programme of research has been granted ethical approval by the National University of Ireland, Galway Ethics Committee (HRB20-Apr-17), the ethical committee of Galway University Hospital (C.A 2366), and the ethical committee of the University of Milano-Bicocca (0047189/20). Written consent will be provided by all participants prior to participation. Both quantitative and qualitative data will be handled in accordance with GDPR guidelines. 

## Study status

Work package 1: Recruitment is in progress and projected to complete February 2021.

Work package 2: Recruitment is currently in progress and projected to complete Feburary 2021.

Work package 3: Start February 2021.

## Discussion

Recent publications have covered a number of initiatives aimed at alleviating psychological distress in FLWs during the COVID-19 pandemic. In Wuhan, a group of researchers have implemented a social media campaign where FLWs can seek social support from one another or from volunteers, exchange advice on alleviating psychological distress, and connect with local and national authorities to express their needs ( Cheng *et al*., 2020). In France, a COVID-19 psychological support hotline was piloted, whereby hospital staff could report their symptoms, receive advice on available support services, and obtain referrals for psychological and psychiatric support if needed ( Geoffroy *et al*., 2020). In the UK, a digital package is available to FLWs which contains information on how to manage and communicate their emotions, create psychologically safe workplaces, and engage in self-care strategies ( Blake *et al*., 2020). However, few psychological support initiatives have been developed using the advised criteria for providing psychological care following mass traumatic events, whereby interventions are based on the identification of unique sources of stress and resilience in those exposed to the traumatic event, perceived barriers to psychological care, and groups at-risk of psychological distress ( Reifels *et al*., 2013). Consequently, the specific psychological requirements of FLWs remain poorly understood. The proposed project will address this issue using a mixed methods approach, whereby best practice guidelines are developed using quantitative and qualitative data, collected from a large, diverse cohort of FLWs, and validated using the Delphi method. Once validated, the guidelines will be disseminated to relevant mental health bodies and provide clinicians with a vital stakeholder-informed framework for alleviating psychological distress in FLWs during the current pandemic, and future public health emergencies.

## Limitations

The main limitation of this research is generalisability, given that only Irish and Italian FLWs will be recruited for participation. Although the COVID-19 pandemic has severely impacted the health services of many countries internationally, the findings may not be applicable to FLWs outside of a European context. However, this is necessary to ensure that the resulting best practice guidelines are suitable for the Irish context, with Italian data offering an additional perspective from a country which has experienced the COVID-19 pandemic differently. Furthermore, although convenience sampling is necessary due to the demands currently faced by FLWs, the lack of random sampling could further reduce the generalizability of the findings. Another potential limitation is the reliance on subjective assessments, either through self-reported questionnaires or semi-structured interviews. As these assessments are susceptible to recall and social desirability biases, both the quantitative and qualitative findings may not accurately reflect the experiences or perceptions of the participants.

## Conclusion

This proposed study will evaluate the proportion of Irish and Italian FLWs reporting various symptoms of psychological distress, and the determinants of these symptoms. This study also intends to qualitatively assess sources of stress, resilience, and barriers to psychological care in FLWs during the different stages of the COVID-19 pandemic. These findings will be used to inform the development of best-practice guidelines for alleviating psychological distress in FLWs, which will be validated using the Delphi method. Although previous research has identified a large proportion of FLWs reporting elevated symptoms of psychological distress during the COVID-19 pandemic, there are currently no guidelines regarding optimal strategies for alleviating these symptoms. Accordingly, this research will address a notable literature gap, and provide clinicians with a vital framework for alleviating psychological distress in FLWs during pandemics.

## Data sharing

We will comply with the Wellcome Trust “Sharing research data and findings relevant to the novel coronavirus (COVID-19) outbreak” statement ( Wellcome Trust, 2020), and share interim and final research findings as well as anonymised data with the public health research community. Final research findings will be submitted for open access publication in line with this statement, and all data sharing procedures will be guided by FAIR Principles ( Wilkinson *et al*., 2016).

## Data availability

No data are associated with this study.
