# Supplementary material for: The identification of needs and development of best practice guidance for the psychological support of frontline healthcare workers during and after COVID-19: A protocol for the FLoWS project
Source: HRB Open Res. 2021 Jan 29;3:54. Originally published 2020 Aug 17. [Version 2] doi: 10.12688/hrbopenres.13117.2 (PMC8030118; doi:10.12688/hrbopenres.13117.2)
Supplement: Peer review history

# Peer review: Reviewer response for version 2

**DOI:** 10.21956/hrbopenres.14379.r28809

**Published:** 2021-04-07

**Authors:** Guendalina Graffigna

**Affiliations:** 1 Faculty of Agriculture, Food and Environmental Sciences, Università Cattolica del Sacro Cuore, Piacenza, Italy

I have read and assessed the manuscript and I think it may be approved now.

Is the study design appropriate for the research question?

Partly

Is the rationale for, and objectives of, the study clearly described?

Yes

Are sufficient details of the methods provided to allow replication by others?

Yes

Are the datasets clearly presented in a useable and accessible format?

Not applicable

Reviewer Expertise:

NA

I confirm that I have read this submission and believe that I have an appropriate level of expertise to confirm that it is of an acceptable scientific standard.

# Peer review: Reviewer response for version 2

**DOI:** 10.21956/hrbopenres.14379.r28808

**Published:** 2021-02-01

**Authors:** Gary Lamph

**Affiliations:** 1 Faculty of Health and Wellbeing, University of Central Lancashire, Preston, UK

I only suggested minor corrections previously. I am therefore satisfied with the changes made and feel that this paper is suitable for indexing.

Is the study design appropriate for the research question?

Yes

Is the rationale for, and objectives of, the study clearly described?

Yes

Are sufficient details of the methods provided to allow replication by others?

Yes

Are the datasets clearly presented in a useable and accessible format?

Not applicable

Reviewer Expertise:

Qualitative Researcher and Previous Paper in this are of interest relating to the COVID pandemic. I am less experienced in the WP1 Quantitative analysis sections of this paper and whilst the methods outlined look reasonable I would suggest further clarification from someone which more expertise here.

I confirm that I have read this submission and believe that I have an appropriate level of expertise to confirm that it is of an acceptable scientific standard.

# Peer review: Reviewer response for version 1

**DOI:** 10.21956/hrbopenres.14225.r27837

**Published:** 2020-11-12

**Authors:** Guendalina Graffigna

**Affiliations:** 1 Faculty of Agriculture, Food and Environmental Sciences, Università Cattolica del Sacro Cuore, Piacenza, Italy

The study protocol is interesting and aims at achieving relevant results, which is particularly needed in the current pandemic of COVID-19.

 The main methodological limits of the protocol are the following:

 WP1: in the sampling strategy there is not a clear mention of how different professional categories will be involved and handled. Will the sample be stratified per GP, per specialists of different specialties, per nurses, etc? How will the analysis will be articulated per different HCP categories? Furthermore, the organizational characteristics of the healthcare organizations in which the interviewees are working is not considered in the sampling strategies and in the analysis procedure, although these aspects greatly impact on burnout and HCP’s work experiences. Moreover, no mention is provided about how the geographical and cultural setting in which the interviewees work may impact on their level of stress and burnout. Italy, for instance, is a country where the management of the healthcare services differ across regions. Furthermore, the epidemiological characteristics of the COVID-19 differs among different areas of Italy. Thus all these aspects should be taken into better consideration for structuring the WP1 sample and for conducting better sounds analysis. Finally, it is not clear if the interviewees will have to have experienced directly of treating COVID-19 patients or of working in COVID-19 centers. All these issues would greatly impact on their work related wellness and need to be carefully considered in the planning of WP1.

 WP2: it is not clear how this WP investigation strategy (i.e. sampling strategy, interview guide…) will build on the WP1 results. Particular, the interdependence between WP1 and WP2 should be better clarified and should be reflected in the sampling strategy and in the interview guide. A better articulation of these points is needed.

 WP3: aspects related to feasibility of the psychological support in different typology of healthcare settings needs to be explored and better reported. Furthermore, the authors should clarify how methodologically they plan to handle the issue of cross-organizational and cross-cultural transferability of the guidelines reached in WP3. Aspects related to the economic and organizational sustainability of the service should be addressed as well.

Is the study design appropriate for the research question?

Partly

Is the rationale for, and objectives of, the study clearly described?

Yes

Are sufficient details of the methods provided to allow replication by others?

Yes

Are the datasets clearly presented in a useable and accessible format?

Not applicable

Reviewer Expertise:

NA

I confirm that I have read this submission and believe that I have an appropriate level of expertise to confirm that it is of an acceptable scientific standard, however I have significant reservations, as outlined above.

# Response

**Published:** 2021-02-01

**Authors:** Jack Flynn

**Affiliations:** National University of Ireland, Galway, Ireland

Dr Graffigna,

 Thank you for taking the time to review our protocol and for the suggestions you have given us to improve it. Below, we address your report item-by-item.

*Will the sample be stratified per GP, per specialists of different specialties, per nurses, etc? How will the analysis will be articulated per different HCP categories?*

 The sample will not be stratified, however we do collect data on the participant’s exact role in healthcare and will incorporate this into analysis.

*the organizational characteristics of the healthcare organizations in which the interviewees are working is not considered in the sampling strategies and in the analysis procedure, although these aspects greatly impact on burnout and HCP’s work experiences*

 While this is not directly asked about, anecdotally we have observed organizational structure related comments being made both in the interviews and in a free text question in the online questionnaire. We also have healthcare workers in our steering group who have provided input on organizational considerations, and will continue to do so to help contextualize the data as we create our guidelines. In addition, we hope to recruit a diverse range of healthcare workers to take part in WP3. 

*no mention is provided about how the geographical and cultural setting in which the interviewees work may impact on their level of stress and burnout. Italy, for instance, is a country where the management of the healthcare services differ across regions. Furthermore, the epidemiological characteristics of the COVID-19 differs among different areas of Italy. Thus all these aspects should be taken into better consideration for structuring the WP1 sample and for conducting better sounds analysis.*

 We record participant location and work setting in both the Irish and Italian arms, and plan to incorporate this into the analysis.

*Finally, it is not clear if the interviewees will have to have experienced directly of treating COVID-19 patients or of working in COVID-19 centers. All these issues would greatly impact on their work related wellness and need to be carefully considered in the planning of WP1*

 We agree that this is a major factor in the impact on wellness. We developed a definition of a frontline healthcare worker as “a person working in healthcare whose role during the pandemic was likely to require repeated contact with patients with COVID-19, whether confirmed or suspected”. This was agreed with our steering group as being broad enough to apply across healthcare roles and experience levels while still ensuring that the core requirement of working directly with COVID patients was clear.

*WP2: it is not clear how this WP investigation strategy (i.e. sampling strategy, interview guide…) will build on the WP1 results. Particular, the interdependence between WP1 and WP2 should be better clarified and should be reflected in the sampling strategy and in the interview guide. A better articulation of these points is needed.*

 WPs 1 and 2 are running concurrently, and for the most part share the same sampling strategy which we now explicitly state in the text. The online questionnaire and the interview guide were developed in tandem to complement each other, this has now also been clarified.

*WP3: aspects related to feasibility of the psychological support in different typology of healthcare settings needs to be explored and better reported.*

*[…]*

*Aspects related to the economic and organizational sustainability of the service should be addressed as well.*

 The feasibility of psychological supports in various settings is an important point we now expand on further, by explaining our intention to ensure we have representation of those with the power to implement such supports as well as the “target audience” who we propose will avail of it within the stakeholder panel. Sustainability of such offerings will also be a key consideration; we also predict that distinctions will need to be made when evaluating the sustainability of suggestions for supports meant for short-term use as an immediate reaction to a certain level of pressure on FLWs as opposed to those proposed as longer-term offerings.

*How will the analysis be articulated per different HCP categories?*

 We have clarified that occupational status will be included as a predictor within the logistic regression analyses, and that odds ratios and 95% confidence intervals will be used to determine whether certain occupational groups are at higher odds of experiencing psychological distress.

*Moreover, no mention is provided about how the geographical and cultural setting in which the interviewees work may impact on their level of stress and burnout. Italy, for instance, is a country where the management of the healthcare services differ across regions. Furthermore, the epidemiological characteristics of the COVID-19 differs among different areas of Italy.*

 We have made clearer that the country where the participants reside (i.e., Ireland or Italy) will be included as a predictor variable within the regression analyses, which will indicate whether the residents of either country are at higher odds of experiencing psychological distress. Although it is not feasible to assess whether the specific regions within the countries where the participants reside will influence their likelihood of experiencing psychological distress do to sample size considerations, we have also clarified that the logistic regressions will include the type of region where participants reside (i.e., rural or urban), which can approximate regional differences between each country.  

*Furthermore, the authors should clarify how methodologically they plan to handle the issue of cross-organizational and cross-cultural transferability of the guidelines reached in WP3. Aspects related to the economic and organizational sustainability of the service should be addressed as well.*

 We have clarified that the findings from work packages 1 and 2 will be examined to determine whether they are congruent between different cultures and occupational groups and if the data are not congruent, the guidelines will be developed to reflect these differences. Moreover, the economic and organizational sustainability can be addressed during the Delphi process, whereby experts provide reasons as to why different proposals within the guidelines may not be practical (for example, not being economically feasible).

# Peer review: Reviewer response for version 1

**DOI:** 10.21956/hrbopenres.14225.r28177

**Published:** 2020-11-10

**Authors:** Gary Lamph

**Affiliations:** 1 Faculty of Health and Wellbeing, University of Central Lancashire, Preston, UK

Thank you for submitting this very well written and important protocol that is of current interest and requires timely publication inline with the Global COVID pandemic. The paper provides a clear and concise outline of your protocol and a thorough rationale for the study and the outline work packages (WPs) to be utilised within the protocol.

 I would recommend this paper for indexing but identified some minor changes for consideration. 

 The first sentence of paragraph 3 needs re-writing as does not flow correct. *Those at the healthcare frontline in responding to COVID-19, including doctors, nurses and paramedics, have had to quickly adapt to a novel, highly transmittable, and lethal disease.*

 Within the background section you identify that not much work has been complete in this area. However in the UK a practical support model that has combined other national guidance has been proposed but is untested. Tomlin, J., Dalgleish-Warburton & Lamph G ****(2020) ^1^. This model builds upon national guidelines for supporting the psycho-social support of FLWs during the COVID pandemic with suggestions of individual and organisational responses. Hence I draw your attention to this as it might be useful to consider / include within the context of this study now within this paper, or later in the study. 

 Population - does this only include only FLW in acute adult services, needs to be clearer, what about older persons residential settings, mental health settings, they have their own unique challenges with working with COVID on the frontline but if they are excluded could this be clarified within this section? 

 Work package 1 – I wondered here if this could be enhanced by further clarifying how analysis of data will work given the participants could be from a broad and wide range of services and graphical locations, are there any plans to analyse data in specific services or areas, could this be made clearer? 

 Work package 2 – will the participants come from the same sample of WP1. Do you anticipate a cross over of participants involved across both WPs or a new group of participants for WP2 could you clarify within the paper?

 Work package 2 – You provide a good clear rationale for approaches taken. However how were the research questions developed, could you provide some context and rationale for this? Within Q1 the different stages of COVID are mentioned but you have not previously mentioned these. Tomlin *et al.* (2020) mention COVID stages do they align, or have you aligned these stages of COVID-19 to another publication source? I think you need to provide some clarity about what the different stages are. 

 Framework analysis seems like an excellent choice with its within case and across theme analysis potential. You mention saturation on page 6 just before you move onto discuss WP3. I think this should also be mentioned earlier when you discuss your sample size of WP2 being between 20-30. Will recruitment discontinue once saturation is achieved? 

 Can you update study status to time of publication once approved.

 Overall I think this is an important contribution to the literature in this rapidly changing and emerging area of concern. I look forward to seeing this paper indexed and reading subsequent results outputs.

Is the study design appropriate for the research question?

Yes

Is the rationale for, and objectives of, the study clearly described?

Yes

Are sufficient details of the methods provided to allow replication by others?

Yes

Are the datasets clearly presented in a useable and accessible format?

Not applicable

Reviewer Expertise:

Qualitative Researcher and Previous Paper in this are of interest relating to the COVID pandemic. I am less experienced in the WP1 Quantitative analysis sections of this paper and whilst the methods outlined look reasonable I would suggest further clarification from someone which more expertise here.

I confirm that I have read this submission and believe that I have an appropriate level of expertise to confirm that it is of an acceptable scientific standard.

## References

[1] : Psychosocial Support for Healthcare Workers During the COVID-19 Pandemic. *Front Psychol*.2020;11: 10.3389/fpsyg.2020.01960 1960 10.3389/fpsyg.2020.01960 32849149PMC7431467
